# Male initiation in contraceptive negotiation among Boda-Boda riders in Kampala, Uganda

**DOI:** 10.3389/ijph.2026.1609598

**Published:** 2026-10-19

**Authors:** Kevin Micheal Mugisa, Joseph Rujumba, Li-Yin Chien

**Affiliations:** 1 International Health Program, National Yang Ming Chiao Tung University, Taipei, Taiwan; 2 Department of Paediatrics and Child Health, College of Health Sciences, Makerere University, Kampala, Uganda; 3 Institute of Community Health Care, College of Nursing, National Yang Ming Chiao Tung University, Taipei, Taiwan

**Keywords:** Boda-Boda riders, contraceptive negotiation, male involvement, partner communication, Uganda

## Abstract

**Objectives:**

This study examined male initiation of contraceptive negotiations, related factors, and associations with method agreement and use frequency among Boda-Boda riders motorcycle taxi operators in Uganda.

**Methods:**

A cross-sectional survey (August–September 2023) included 421 sexually active male Boda-Boda riders in Kampala. Logistic and multinomial logistic regression models were used to analyze the data.

**Results:**

Overall, 54.39% of participants initiated contraceptive negotiations, mostly regarding hormonal methods (55%). Initiation was positively associated with age <45 years, history of sexually transmitted infections, recent receipt of family planning information, and partner contraceptive side effects. Factors unexpectedly negatively associated included income ≥1M Ugandan shillings, commercial sex work, and discussing family planning with healthcare workers, the last potentially reflecting reverse causation (men consulting providers after a partner raised the topic). Initiators had significantly higher odds of adopting all contraceptive methods and reporting consistent contraception use.

**Conclusion:**

Results highlight the need for targeted, male-friendly, and couple-centered interventions. Risk-tailored social behavioral change communication practices and inclusive family planning services are recommended to strengthen reproductive health decision-making.

## Introduction

Uganda’s population has grown from 9.5 million in 1969 to 45.9 million in 2024, with a 2.8% annual growth rate [1]. The fertility rate is 5.9 births per woman in rural areas and 4.2 in urban areas, compared to 1.7–1.8 globally [2]. Although many citizens are aware of contraceptives [3], their uptake is low, at 39% [4]. Factors such as education, age, wealth, fear of side effects, residence, service quality, alcohol consumption, income, sex, and early sexual activity influence contraceptive use [5]. Despite efforts, regional differences persist in Uganda. For example, the Karamoja region has a high fertility rate (7.9 children per woman), maternal death (588 per 100,000 live births), and contraceptive non-use (92.7%) among married women aged 15–49 years [6].

Contraception lowers maternal and infant mortality [7], boosts reproductive autonomy [8], fosters economic growth [9], and reduces the number of Human Immunodeficiency Virus (HIV)-positive children [10]. Male initiation in contraceptive negotiations, proactive engagement in discussions, and support for sexual partners are crucial for shared reproductive health decisions [11] and for promoting gender equity in a patrilineal society [12]. However, traditional male-focused methods have been replaced by modern techniques for women, reducing male participation despite their influential role in decision-making, which can affect male initiation [13].

A man’s life stage, whether a teenager, newly married, or a first-time father, affects his sexual needs and reproductive health concerns [14]. Men fulfil their duties and expectations while exhibiting habits that influence romantic relationships and contraceptive negotiations [12]. Typical behavioral goals of male initiation include using contraceptives with partners and engaging in open communication and decision-making about family planning [15], promoting gender equity in families and communities [16]. Male-involvement programs have shown the potential to reduce unwanted pregnancies and HIV infections while increasing healthcare access [17]. However, these programs face obstacles such as low awareness and harmful gender stereotypes [18]. Effective participation programs can change men’s attitudes and behaviors regarding reproductive health [19].

Targeted interventions such as men-only dialogues, community and radio talks, youth engagement, and training leaders have successfully involved men in sexual reproductive health and rights services (SRHR) [20]. These efforts have improved attitudes toward family planning, increased clinic visits, and enhanced contraception discussions. However, gaps remain, particularly in the understanding of male initiation among “Boda-Boda” riders. Boda-Boda is a type of motorcycle with a space for a passenger or for carrying goods, often used as a taxi [21]. Boda-Boda riders are characterized by high mobility, frequent rural–urban migration, and complex sexual networks. These networks encompass diverse groups, including adults, widows, youths, college students, “sugar mummies,” waitstaff, sex workers, and fashion designers, highlighting the unique challenges in promoting effective family planning strategies within this community. Boda-Boda riders also travel with money to attract women, use drugs and alcohol, and have various sexual relationships, making the population a priority target [21].

Several studies have been conducted on contraceptive negotiation [22]. Factors that influence contraceptive negotiations include marital status, ethnic diversity, financial instability, multiple partners, income, dependents, education, health conditions, alcohol consumption, residence, mobility, home return times, household type, age, reproductive goals, and access to contraceptives [23]. However, these studies emphasized women [24] and married couples and were qualitative studies [25]. Besides, these studies assessed condom negotiation [26], which is not the entire spectrum of contraceptive negotiations. There is a knowledge gap regarding men as the initiators of contraceptive negotiations. This study addresses critical gaps by examining male initiation in contraceptive negotiations among Boda-Boda riders, offering valuable data to inform sexual reproductive health and targeted interventions in Uganda. This study examined male initiation of contraceptive negotiations and related factors, and associations between initiation and both method agreement and frequency of use among Boda-Boda riders.

## Methods

### Design and participants

This cross-sectional questionnaire-based survey was conducted at 579 approved Boda-Boda stages in Kampala’s five divisions (Central, Kawempe, Makindye, Nakawa, and Rubaga). Eligible participants were men who had worked for at least 6 months, had a female sexual partner in the past 3 months, were aged >18 years, and were proficient in English or Luganda.

The target population comprised 25,226 registered boda-boda riders across Kampala’s five divisions, as obtained directly from Kampala Capital City Authority offices [27]. Prior estimates were segregated as 8,460, 1,276, 7,604, 4,239, and 3,647 for Rubaga Division, Makindye Division, Kawempe Division, Nakawa Division, and Central Division, respectively. The required sample size was 421, determined using Cochran’s (1977) sample size calculation formula, considering 50% prevalence, a 5% margin of error, and a 10% non-response rate. Due to a lack of prior studies, we assumed 50% prevalence because 50% is the “worst-case scenario” for sample size calculation, which means it requires the largest sample size. Convenience sampling with proportional representation using the proportion allocation from each of the five divisions (Central = 61, Kawempe = 126, Makindye = 21, Nakawa = 71, and Rubaga = 142) was planned. Recruitment yielded 421 participants across the same five divisions, with slight differences, as one participant shifted from Makindye to Central (Central = 62, Makindye = 20; see Table 1). The first author or one of the five trained research assistants approached the participants in each division, explained the study, assessed eligibility, obtained written consent, and conducted the interview.

**TABLE 1:** Characteristics of the participants by male initiation of contraceptive negotiation in the last 3 months (N = 421) (Male initiation in contraceptive negotiation study, Uganda, 2023).

Variable	n (%)	Contraceptive negotiation initiation		
		Yes (%)	No (%)	*p*
		n = 229	n = 192	
Age				0.017
18–24	40 (9.5)	20 (50.0)	20 (50.0)	​
25–34	167 (39.67)	106 (63.47)	61 (36.53)	
35–44	164 (38.95)	82 (50.0)	82 (50.0)	
≥45	50 (11.88)	21 (42.0)	29 (58.0)	
Education level				0.363+
No formal education	7 (1.66)	4 (57.14)	3 (42.86)	​
Primary	205 (48.69)	105 (51.22)	100 (48.78)	​
Secondary	200 (47.51)	113 (56.50)	87 (43.50)	​
Tertiary	9 (2.14)	7 (77.78)	2 (22.22)	​
Marital status				0.55
Single	26 (6.18)	17 (65.38)	9 (34.62)	​
Cohabiting	201 (47.74)	110 (54.73)	91 (45.27)	​
Married	183 (43.47)	95 (51.91)	88 (48.09)	​
Separated/divorced/widowed	11 (2.61)	7 (63.64)	4 (36.36)	​
Religious background				0.708
Catholic	142 (33.73)	81 (57.04)	61 (42.96)	​
Protestant	121 (28.74)	61 (50.41)	60 (49.59)	​
Muslim	108 (25.65)	57 (52.78)	51 (47.22)	​
Born again	33 (7.84)	19 (57.58)	14 (42.42)	​
Other	17 (4.04)	11 (64.71)	6 (35.29)	​
Tribe				0.253+
Baganda	245 (58.19)	137 (55.92)	108 (44.08)	​
Basoga	18 (4.28)	8 (44.44)	10 (55.56)	​
Bagisu	10 (2.38)	6 (60.0)	4 (40.0)	​
Banyankole	74 (17.58)	32 (43.24)	42 (56.76)	​
Bakiga	11 (2.61)	7 (63.64)	4 (36.36)	​
Others	63 (14.96)	39 (61.90)	24 (38.10)	​
Urbanicity of residence				0.621+
Urban (kampala)	107 (25.42)	56 (52.34)	51 (47.66)	​
Peri-urban	314 (74.58)	173 (55.10)	141 (44.90)	​
Distance between home and work (km). M(SD)	7.4 (12.9)	7.9 (15)	6.7 (9.4)	0.327
Division of the boda-boda stage				<0.001
Nakawa	71 (16.86)	55 (77.46)	16 (22.54)	​
Rubaga	142 (33.73)	81 (57.04)	61 (42.96)	​
Kawempe	126 (29.93)	64 (50.79)	62 (49.21)	​
Central	62 (14.73)	24 (38.71)	38 (61.29)	​
Makindye	20 (4.75)	5 (25.0)	15 (75.0)	​
Number of biological children				0.049
0	30 (7.13)	16 (53.33)	14 (46.67)	​
1	46 (10.93)	27 (58.70)	19 (41.30)	​
2	76 (18.05)	40 (52.63)	36 (47.37)	​
3	78 (18.53)	47 (60.26)	31 (39.74)	​
4	78 (18.53)	40 (51.28)	38 (48.72)	​
5	34 (8.08)	23 (67.65)	11 (32.35)	​
6	31 (7.36)	20 (64.52)	11 (35.48)	​
≥7	48 (11.40)	16 (33.33)	32 (66.67)	​
Income per month (UGX)				<0.001
<500,000	95 (22.84)	65 (68.42)	30 (31.58)	​
500,000–1Million	209 (50.24)	121 (57.89)	88 42.11)	​
≥1Million	112 (26.92)	40 (35.71)	72 (64.29)	​
STD/STId diagnosis				0.003
Yes	159 (37.77)	101 (63.52)	58 (36.48)	​
No	262 (62.23)	128 (48.85)	134 (51.15)	​
HIVe				0.051
Yes	7 (1.66)	1 (14.29)	6 (85.71)	​
No	414 (98.34)	228 (55.07)	186 (44.93)	​
Menopausal sexual partner				0.002+
Yes	7 (1.67)	2 (28.57)	5 (71.43)	​
No	394 (93.81)	222 (56.35)	172 (43.65)	​
I Do not know	17 (4.05)	3 (17.65)	14 (85.35)	​
I Do not want to tell	2 (0.48)	1 (50.0)	1 (50.0)	​
Alcohol consumption				0.059
Yes	155 (36.82)	75 (48.39)	80 (51.61)	​
No	266 (63.18)	154 (57.89)	112 (42.11)	​
Sexual partner’s alcohol consumption				0.94
Yes	73 (17.34)	40 (54.79)	33 (45.21)	​
No	348 (82.66)	189 (54.31)	159 (45.69)	​
Number of sexual partners in the last 3 months				0.669
1	284 (68.77)	157 (55.28)	127 (44.72)	​
2	81 (19.61)	47 (58.02)	34 (41.98)	​
3	27 (6.54)	12 (44.44)	15 (55.56)	​
≥4	21 (5.08)	12 (57.14)	9 (42.86)	​
Received FPf information in the last 3 months				<0.001
Yes	377 (89.55)	218 (57.82)	159 (42.18)	​
No	44 (10.45)	11 (25.0)	33 (75.0)	​
Source of contraceptive advice (among those who received FP information.)	​	​	​	<0.001
None	44 (10.45)	11 (25.0)	33 (75.0)	​
Health center	116 (27.55)	78 (67.24)	38 (32.76)	​
Media	202 (47.98)	106 (52.48)	96 (47.52)	​
Friend	59 (14.01)	34 (57.63)	25 (42.37)	​
Selling or buying sex				0.004
Yes	153 (36.34)	69 (45.10)	84 (54.90)	​
No	268 (63.66)	160 (59.70)	108 (40.30)	​
Easy to access family planning services				0.719
Yes	405 (96.2)	221 (54.57)	184 (45.43)	​
No	16 (3.8)	8 (50.0)	8 (50.0)	​
Easy for a sexual partner to access family planning services				0.493
Yes	392 (93.11)	215 (54.85)	177 (45.15)	​
No	29 (6.89)	14 (48.28)	15 (51.72)	​
Discussed family planning with a healthcare worker				<0.001
Yes	253 (60.1)	99 (39.13)	154 (60.87)	​
No	168 (39.9)	130 (77.38)	38 (22.62)	​
Contraceptive method side effects (self)				0.978
Yes	24 (5.76)	13 (54.17)	11 (45.83)	​
No	393 (94.24)	214 (54.45)	179 (45.55)	​
Contraceptive method side effects (partner)				<0.001
Yes	173 (41.39)	116 (67.05)	57 (32.95)	​
No	245 (58.61)	112 (45.71)	133 (54.29)	​
Ownership of boda-boda				0.936
Yes	293 (69.6)	159 (54.27)	134 (45.73)	​
No	128 (30.4)	70 (54.69)	58 (45.31)	​
Average distance driving per day (km)				0.793
5–50	73 (17.46)	37 (50.68)	36 (49.32)	​
51–100	201 (48.09)	110 (54.73)	91 (43.27)	​
101–150	94 (22.49)	55 (58.51)	39 (41.49)	​
≥151	50 (11.96)	27 (54.0)	23 (46.0)	​
Living outside of home per week				0.506
Yes	50 (11.88)	25 (50.0)	25 (50.0)	​
No	371 (88.12)	204 54.99)	167 (45.01)	​
Contraceptive method agreements				<0.001
No method	77 (18.29)	12 (15.58)	65 (84.42)	​
Barrier method	57 (13.54)	24 (42.11)	33 (57.89)	​
Hormonal method	233 (55.34)	164 (70.39)	69 (29.61)	​
Natural method	46 (10.93)	23 (50.0)	23 (50.0)	​
Traditional method	8 (1.9)	6 (75.0)	2 (25.0)	​
Frequency of contraceptive use				<0.001
Always	336 (79.81)	210 (62.50)	126 (37.50)	​
Sometimes	18 (4.28)	8 (44.44)	11 (55.56)	​
Never	67 (15.91)	11 (16.42)	56 (83.58)	​

P-values were calculated using independent-samples t-tests or chi-square tests. + p-values from Fisher’s exact test. a M(SD): mean (standard deviation); b USD: US, dollars; c UGX: ugandan shillings; d STD/STI: sexually transmitted disease/sexually transmitted infection; e HIV: human immunodeficiency virus; f FP: family planning; 1USD, 3,776.47 UGX, as of May 27, 2026. Missing values: income (n = 5), menopausal status of partner (n = 1), number of sexual partners (n = 8), contraceptive side effects (self) (n = 4), contraceptive side effects (partner) (n = 3), and average distance driving (n = 3). The 7 HIV-positive cases are included within the STD/STI, diagnosis category (n = 159). Percentages for each variable are based on the number of respondents with non-missing data for that variable (i.e., denominators are the full N = 421 unless a specific missing count is noted for that variable). Source of contraceptive advice was only asked of participants who reported receiving family planning information in the last 3 months (n = 377); the ‘None’ category corresponds to the 44 participants who did not receive family planning information.

The participants were interviewed using a structured questionnaire. Data was collected from August to September 2023 (422 out of 430 approaches; response rate = 98%), and one participant was excluded because of inconsistent responses (final sample size = 421). Confidential interviews were conducted in English or Luganda, and the supervisor checked the questionnaires daily.

This study was approved by the Institutional Review Boards of the National Yang Ming Chiao Tung University, Makerere University School of Public Health, and the Uganda National Council for Science and Technology (reference numbers: NYCU112121AE, SPH-2023-478, and HS310ES, respectively). We also Obtained local authorization from the Kampala Capital City Authority (reference numbers: DPHE/KCCA/201/22 and MKD/KCCA/317).

### Measures

Data collection tools were pretested with 43 Boda-Boda riders, resulting in adjustments. Initiation of contraceptive negotiations was measured by asking a Yes/No question: In the last 3 months, did you start a conversation about contraceptive use with your sexual partner/partners? Method agreements were categorized into barrier, hormonal, natural, and traditional. The frequency of contraceptive use was categorized as always, sometimes, or never. Factors related to contraceptive initiation were grouped into socio-demographics (age, education level, marital status, religious background, tribe, urbanicity, distance between home and work, Boda-Boda stage division, number of biological children, commercial sex, and income). Health behavioral status included respondents’ health status [having a sexually transmitted disease or infection (STD or STI), including HIV] and sexual partners’ health status (if they had HIV or were experiencing menopause). Other health behavioral factors included alcohol consumption, number of sexual partners in the last 3 months, contraceptive advice source, discussions about family planning with healthcare workers, engagement in commercial sex, accessibility to contraceptives, and experiences with contraceptive side effect. Contextual and work-related factors were assessed through ownership of a boda-boda, average distance driven per day, and number of nights spent outside the home per week.

### Data analysis

The data were initially entered into Microsoft Excel and subsequently imported into STATA version 15 [28] for statistical analysis. Descriptive statistics were used to summarize the key variables. Continuous variables were assessed using independent sample t-tests to compare means and standard deviations, whereas categorical variables were evaluated using chi-squared tests to assess differences in proportions. Logistic regression analyzed the factors associated with negotiation initiation. Respondents’ HIV status was not modeled independently due to sparse data (n = 7). HIV positive cases were retained within the composite STD/STI diagnosis variable used in the regression model. We combined categories with small sample size in the modelling. Age was dichotomized at 45 years because the bivariate relationship was non-monotonic (with a peak in the 25–34 group and lower proportions in both younger and older groups), and the 45-year cut-off separated riders in the family-formation years from those past them while preserving statistical power.

The final parsimonious model was developed using multivariable logistic regression with backward elimination. Variables significant at p < 0.10 in bivariate analysis were entered into the initial multivariable model. Non-significant variables (p > 0.05) were sequentially removed using likelihood ratio tests. A variable was retained in the final model if it remained statistically significant, or if its removal changed the coefficients of remaining variables by more than 20% (indicating confounding), or if its removal worsened model fit based on AIC/BIC criteria. Model fitness was evaluated using the Hosmer- Lemeshow test. Multinomial logistic regression was used to examine the association between contraceptive negotiation initiation, method agreements, and frequency of contraception use. Crude odds ratios (CORs) were calculated to measure the strength of the associations, with statistical significance determined using a p-value threshold of <0.05.

## Results

The analytic sample included 421 male boda-boda riders, with a mean age of 35.04 years (SD = 7.95). Most had completed primary (48.7%) or secondary (47.5%) education, and most were cohabiting (47.7%) or married (43.5%). Religious affiliation was predominantly Catholic (33.7%) and Protestant (28.7%), and the largest tribal group was Baganda (58.2%). Average income was 814,663 Uganda shillings (SD = 367,246; approximately 236 USD). Sample characteristics are reported in Table 1.

In the bivariate analysis, of the 421 respondents, 229 (54.4%) reported initiating contraception negotiation with a sexual partner in the last 3 months, and the comparisons identified several statistically significant factors related to initiation (Table 1). Age was associated with contraception negotiation initiation (p = 0.017); the 25-34 age group had the highest proportion of initiators (63.5% versus 50% for 18-24 and 35-44; 42% for ≥45). Initiation also varied by boda-boda stage division, ranging from 77.5% in Nakawa to 25.0% in Makindye (p < 0.001). Initiators had fewer biological children than non-initiators (3.7 versus 4.3, p = 0.049). Initiators earned less on average than non-initiators (748,982 Ugandan shillings Vs. 892,790 Uganda shillings; p < 0.001). Those with STD/STI diagnosis were more likely to be initiators than without (63.5% versus 48.9%; p = 0.003). Of the 7 participants with HIV, only 1 was an initiator (14.29% versus 55.07% for non-HIV; p = 0.051). Those received family planning information in the past 3 months were more likely to be initiators (57.8% versus 25%, p < 0.001), but those discussed family planning with a healthcare worker were less likely to be initiators (39.1% versus 77.4%, p < 0.001). Partners experienced with contraceptive method side effects were more likely to be initiators (67.05% versus 45.71%, p < 0.001). Those who reported selling or buying sex were less likely to be initiators (45.1% versus 59.7%, p < 0.001).

The final multivariable logistic regression model included seven predictors (Table 2). The model demonstrated good fit (LR χ^2^ (7) = 116.33, p < 0.001; Pseudo R^2^ = 0.202). The Hosmer-Lemeshow goodness-of-fit test was not significant (χ^2^ (7) = 15.43, p = 0.051), indicating that the model adequately fits the data. Collinearity diagnostics showed no evidence of multicollinearity (all VIF <2). After adjustment, men aged <45 years had significantly higher odds of initiating contraceptive negotiation compared to those aged ≥45 years (AOR = 2.66, 95% CI: 1.33–5.31, p = 0.006). Men with income ≥1M had lower odds of initiation compared to those with income <1M (AOR = 0.45, 95% CI: 0.27–0.75, p = 0.002). STD/STI diagnosis (AOR = 1.89, 95% CI: 1.18–3.01, p = 0.008), receipt of family planning information in the last 3 months (AOR = 2.67, 95% CI: 1.22–5.86, p = 0.014), and partner experiencing contraceptive side effects (AOR = 2.27, 95% CI: 1.44–3.58, p < 0.001) were associated with higher odds of initiation. Discussion of family planning with a healthcare worker was associated with lower odds of initiation (AOR = 0.20, 95% CI: 0.12–0.32, p < 0.001). Selling or buying sex was also associated with lower odds of initiation (AOR = 0.58, 95% CI: 0.36–0.94, p = 0.026).

**TABLE 2:** Logistic regression model for contraceptive negotiation initiation (Male initiation in contraceptive negotiation study, Uganda, 2023).

Variable	Odds ratio (95% CI)	P-value
Age <45 years (ref: 45+)	2.66 (1.33–5.31)	0.006
Income ≥1M (ref: <1M)	0.45 (0.27–0.75)	0.002
STD/STI diagnosis (ref: no)	1.89 (1.18–3.01)	0.008
Received information on FP in the last 3 months (ref: no)	2.67 (1.22–5.86)	0.014
Sexual partner experienced contraception side effects (ref: no)	2.27 (1.4–3.6)	<0.001
Discussed family planning with a healthcare worker (ref: no)	0.20 (0.12–0.32)	<0.001
Selling or buying sex (ref: no)	0.58 (0.36–0.94)	0.026

aOR, adjusted odds ratio; CI, confidence interval; Ref = reference category. Variables were selected using backward elimination. Model fit: LR, χ^2^ (7) = 116.33, p < 0.001; Pseudo R^2^ = 0.202; Hosmer-Lemeshow χ^2^ (8) = 15.43, p = 0.051; mean VIF, 1.05.

Multinomial logistic regression analyses showed that contraceptive negotiation initiation was significantly associated with agreement on barrier (COR = 3.9, 95% CI: 1.75–8.80, p < 0.001), hormonal (COR = 12.8, 95% CI: 6.54–25.33, p < 0.001), natural (COR = 5.41, 95% CI: 2.32–12.60, p < 0.001), and traditional methods (COR = 16.25, 95% CI: 2.90–90.20, p < 0.001), as well as always (COR = 8.48, 95% CI: 4.28–16.79, p < 0.001) and sometimes (COR = 4.07, 95% CI: 1.31–12.63, p = 0.015) using contraception (Tables 3, 4).

**TABLE 3:** Multinomial logistic regression analysis of the association between initiation of contraceptive negotiation and contraceptive method agreement (Male initiation in contraceptive negotiation study, Uganda, 2023).

Independent variable	Contraceptive negotiation method agreements			
Contraceptive negotiation initiation (ref: no method)	Barrier method [odds ratio (95% CI)]	Hormonal method [odds ratio (95% CI)]	Natural method [odds ratio (95% CI)]	Traditional method [odds ratio (95% CI)]
Yes	3.9 (1.75–8.8)	12.8 (6.54–25.33)	5.41 (2.32–12.60)	16.25 (2.9–90.2)
P-value	<0.001	<0.001	<0.001	<0.001

Note. OR, odds ratio; COR, crude odds ratio; CI, confidence interval; Ref = reference category.

**TABLE 4:** Multinomial logistic regression analysis of the association between the initiation of contraceptive negotiation and frequency of contraception use (Male initiation in contraceptive negotiation study, Uganda, 2023).

Independent variable	Frequency of contraceptive use	
Contraceptive negotiation initiation (ref: Never)	Always [odds ratio (95% CI)]	Sometimes [odds ratio (95% CI)]
Contraceptive negotiation initiation (ref: Never)	Always [odds ratio (95% CI)]	Sometimes [odds ratio (95% CI)]
Yes	8.48 (4.28–16.79)	4.07 (1.312–12.63)
P-value	<0.001	0.015

OR, odds ratio; COR, crude odds ratio; CI, confidence interval; Ref = reference category.

## Discussion

Over half (54.4%) of the respondents reported initiating contraceptive discussions in the past 3 months. In comparison, studies from the United States indicated that 45% of women were primarily responsible for contraceptive decisions and 55% shared responsibility [29], whereas a meta-analysis showed that men are more likely to initiate negotiations than women [30] in traditional, masculine settings compared to women who are equally or more likely to initiate negotiations in social or domestic contexts [31]. Additionally, a systematic review and meta-analysis in Ethiopia found a 59.7% pooled prevalence of male involvement in family planning. This involvement was defined as participation in at least one activity such as contraceptive negotiation, support, approval, or direct use [32, 33]. Direct comparisons of our results to literatures are limited because male involvement encompasses multiple dimensions of participation; while this study specifically examines whether male initiated contraception negotiations. However, we believed male initiation is an important aspect of contraceptive negotiations, which is supported by our findings that the initiators were more likely to adopt contraceptive methods and report always using contraception.

Men aged <45 years were more likely to initiate negotiations than men aged >45 years, perhaps reflecting life stage (active family formation) [34] and active fertility planning, and greater digital exposure to family planning messaging among younger adult men [35]. A history of STDs and recent receipt of family planning information were also associated with an increased likelihood of negotiation initiation, suggesting that personal or couple risk awareness may be associated with communication. Studies have shown that couples who discuss their sexual health are far more likely to use contraceptives. In Senegal, joint discussions were associated with increased contraceptive use by 56%, and even one partner’s discussion was associated with an increase of 25% [36]. Similar links between spousal communication and increased modern contraceptive use have been found in other African countries [36–38]. Similarly, men whose partners experienced contraceptive side effects were more likely to initiate discussions, presumably to address the suitability of the method. For example, studies from Rwanda found that male partners supported their female partners’ contraceptive use by engaging in discussions about switching methods when side effects became unbearable [39].

Similarly, studies in Uganda, Zambia, and South Africa reported that men’s awareness of side effects (such as irregular bleeding, reduced sexual pleasure, or decreased libido) was associated with participation in conversations about contraceptive choices, either to seek alternatives or address their concerns [40–43]. Programs should leverage the following triggers: sexual health and STI education can explicitly link disease prevention with contraception, and male-inclusive counselling on side effect management can steer couples toward appropriate method choices. Though HIV is part of STI/STD, in bivariate analysis, HIV was negatively associated with initiation of negotiation (p = 0.051), unlike STI/STD was positively associated with initiation. There could be stigma-related avoidance among HIV-positive individuals. But due to the limited number of HIV-positive persons, no further analysis is possible. Future studies are needed to address the potential differences.

Unexpectedly, participants who reported discussing family planning with healthcare workers were less likely to initiate partner negotiations. This contrasts with national survey data. For example, a Ugandan study found that men who discussed family planning with healthcare providers had nearly double the odds of using modern contraception [42]. This could be because men may only seek healthcare worker advice after a partner has already experienced contraceptive side effects [39] or because services may still feel woman-centered so that men may consult passively [42]. There may be cause and consequence confusion in that those men who did not initiate contraceptive negotiation because their partners had involved them in discussing family planning with healthcare workers. The implication here is that male initiation is a behavior associated with social, observational, gendered, and community-driven contexts, with Boda-Boda riders likely to have alternative safe spaces to talk about family planning. In addition, personal introspection and continuous media influence may account for positive behavior and attitudes towards contraceptive negotiation. These insights should further be explored in future studies. Policies and programs should train providers in men-inclusive counseling and outreach (e.g., at Boda-Boda stages) to encourage joint decision-making.

This study showed that high-income participants and those involved in transactional sex were less likely to initiate contraceptive negotiations, perhaps reflecting different norms or relationship types. In comparison, studies in rural South Africa showed that women with higher economic contributions had lower odds of engaging in transactional sex but higher odds of multiple sexual partnerships, suggesting that financial autonomy and relationship status shape sexual negotiations and risky behaviors [43]. In transactional sex contexts, financial incentives and power imbalances are associated with a lower likelihood of negotiating condom use, as seen among male sex workers and their clients in Uganda and South Africa, where higher pay for sex without condoms and economic vulnerability were associated with lower risk reduction [44, 45]. Norms such as “I can afford the family since I earn more” among wealthier men can reinforce male authority and reduce contraceptive negotiation, as shown in India and Pakistan, where restrictive male attitudes and authority norms are negatively associated with women’s contraceptive use, regardless of women’s education or wealth [13, 46]. Interventions that address these authority norms and emphasize risk reduction, such as promoting condom use and gender-equitable attitudes, are recommended to support more equitable negotiations and safer sex practices in key and priority populations, such as Boda-Boda riders.

Respondents who initiated contraception conversations was associated with higher contraceptive uptake and adherence. They were far more likely to report agreeing on hormonal, barrier, or other methods and to say they “always” used contraception. However, some initiators settled on traditional or natural methods, and approximately 15% of the initiators chose less effective methods. Given riders’ high STI risk, programs should emphasize dual protection (e.g., condoms plus a reliable female-controlled method) and counseling on effective options. In general, our findings align with the broader evidence that partner communication drives contraceptive use. For example, women in multiple sub-Saharan African countries who frequently discuss contraception with their partners have higher odds of using a contraceptive method [42]. Similarly, interventions engaging men often boost the use of modern contraceptives. Conversely, entrenched gender norms still portray pregnancy prevention as a woman’s responsibility. Qualitative studies have noted that many young men expect to play only a “partial role” (e.g., sharing costs or initiating a discussion) and see contraception largely as a woman’s responsibility [47]. Male-oriented education, couple counseling, and expanded contraceptive options have been associated with attitude change in some studies [47, 48]. These findings suggest that male participation in contraceptive discussions warrants attention in intervention design, though whether such interventions would increase method adoption or reduce unplanned pregnancies and STI transmission remains to be tested.

### Strengths and limitations

This study provides new insights into male initiation of contraceptive negotiations in a high-risk population. However, several limitations must be acknowledged. The cross-sectional design of this study implied that causality could not be inferred. We did not collect partner type and negotiation dynamics, which may differ between primary and casual sexual encounters, leading to ambiguity. Contraception negotiation initiation was measured using a single self-reported item. As a result, the measure may not fully capture the complexity of behaviors involved in initiating contraception negotiations.

All data were self-reported, so sexual behavior and contraceptive use were subject to recall errors and social desirability bias, which we attempted to mitigate via anonymity and trust-building. We used a convenience sample from registered Boda-Boda stages, which may not represent all riders. Registered Boda-Boda may have more stable relationships than unregistered Boda-Boda riders, who may exhibit more erratic sexual behaviors and have more transient or higher-risk sexual networks; such riders may therefore be underrepresented in this study, limiting generalizability of findings to the highest-risk segments of the rider population that the introduction references.

Some estimates, i.e., traditional methods, were based on small cell counts, resulting in wide confidence intervals and potentially unstable estimates. The small number of HIV-positive participants (n = 7) precluded independent examination of HIV status as a predictor of contraception negotiation initiation. HIV positive cases were therefore included within the composite STD/STI diagnosis variable; therefore, the independent contribution of HIV status could not be assessed in this study.

### Conclusion

This study provides a quantitative high-risk profile of male contraception negotiation initiation among Boda- Boda riders, showing that STI history, recent family planning information exposure, and partner-reported side effects drive initiation while prior contact with a healthcare provider does not. Family planning services should therefore extend outreach directly to Boda-Boda riders and retrain providers to engage male clients on reproductive health decision-making in this mobile and high-risk population.

## Data Availability

The raw data supporting the conclusions of this article will be made available by the authors, without undue reservation.
